# Cytomorphological changes of oral mucosal cells among smokeless tobacco users in low and middle-income country settings: new findings from Pakistan

**DOI:** 10.1186/s12903-024-05220-7

**Published:** 2024-12-23

**Authors:** Sabreen Hassan, Ayesha Sifat, Mohammad Munib, Saleha Saeed, Waqar U. Nisa, Sofia Haider Durrani, Abid Rahim, Naeem Ullah, Saima Afaq, Farhad Ali Khattak, Zia Ul Haq

**Affiliations:** 1 Present Address: Saidu College of Dentistry, Swat, Pakistan; 2 Oral Pathology Department, Saidu College of Dentistry, Saidu Sharif, Swat, Pakistan; 3 Khyber Medical University, Peshawar, Pakistan; 4 Swat Medical College, Swat, Pakistan; 5 Dental College, HITEC – IMS, Taxila, Pakistan; 6 Bacha Khan Dental College, Mardan, Pakistan; 7 Oral Pathology Department, Bacha Khan College of Dentistry, Mardan, Pakistan; 8 Oral Pathology Department,Sardar Begum Dental College, Gandhara University, Peshawar, Pakistan; 9 Gandhara University, Peshawar, Pakistan; 10 Saidu Medical College, Swat, Pakistan; 11 Department of Health Sciences, University of York, York, UK; 12 Institue of Public Health & Social Sciences(IPH&SS), Khyber Medical University(KMU), Peshawar, Pakistan; 13 R&D Cell, Khyber College of Dentistry, Peshawar, Pakistan; 14 School of Health & Wellbeing, University of Glasgow, Glasgow, Scotland, UK

**Keywords:** Oral lesions, Smokeless tobacco, Naswar/snuff, Cellular alterations, Micronuclei, Oral cancer

## Abstract

**Background:**

Chronic tobacco use, in any form, induces significant cellular alterations in the oral mucosa. This study investigates four distinct cytomorphological changes in oral mucosal cells among smokeless tobacco users, examining their association across different genders and age groups.

**Materials and methods:**

This cross-sectional study involved collecting mucosal samples from smokeless tobacco (naswar/snuff) users through consecutive sampling. The prepared smears were examined for dysplastic changes. Data analysis was performed using SPSS, with Chi-square tests and logistic regression employed to evaluate proportions and associations.

**Results:**

Among 100 Naswar/snuff users, the labial sulcus mucosa was the most common site affected (47%). The most frequent cytomorphological change was micronuclei (46%), followed by nuclear budding (25%), perinuclear halo (18%), and binucleated cells (14%). In the 51–60 age group, micronuclei (78.9%), nuclear budding (55.3%), binucleation (31.6%), and perinuclear halo (36.8%) were more prevalent (*P* < 0.005). Gender analysis revealed that micronuclei were more common in females (54.2%) compared to males (43.4%), while nuclear budding was more prevalent in males (27.6%) than females (*P* < 0.28). Logistic regression indicated that individuals aged 51–60 were more likely to exhibit micronuclei (OR = 1.15, 95% CI: 0.22 to 5.83, *P* = 0.863) and nuclear budding (OR = 15.34, 95% CI: 9.23 to 30.75, *P* < 0.05).

**Conclusion:**

The dysplastic changes observed included micronuclei, nuclear budding, binucleated cells, and perinuclear halo, with micronuclei being the most prevalent. These findings could facilitate the early diagnosis of oral lesions and their timely management in habitual smokeless tobacco users.

## Introduction

Oral cancer, predominantly squamous cell carcinoma (SCC), represents one of the most significant health challenges worldwide. The etiology of oral cancer (OC) is complex, with genetic, environmental, social, and behavioral factors playing crucial roles. Among these, tobacco and alcohol consumption are the primary risk factors, contributing significantly to the disease's development. Environmental factors such as alcohol, ultraviolet radiation, and tobacco products damage DNA, increase p53 expression and activate gene clusters associated with cell growth and death. All regions of the oral cavity, including the tongue, roof of the mouth, gums, and cheeks, are susceptible to cancer from tobacco use [1, 2].

According to WHO fact sheets, tobacco consumption by the global population was 22.3%, with 36.7% male population followed by female population 7.8% [3] while other estimates showed that approximately 25.4 million Pakistani adult population consumed tobacco products in 2022, placing Pakistan at 7th position globally in tobacco product users [4]. In the Khyber Pakhtunkhwa, Naswar/snuff is a prevalent form of smokeless tobacco (60%) used by the majority of the population [5]. Naswar/snuff contains over three hundred carcinogenic agents, including nicotine and nitrosamines, which can induce dysplasia and contribute to carcinogenesis. The carcinogenic potential of tobacco is influenced by the quantity, frequency, and duration of use. Early histological changes from tobacco use range from hyper-parakeratosis to epithelial dysplasia. It often takes 20 to 50 years of continuous smokeless tobacco use to initiate malignant changes in the oral mucosa [6, 7]. Despite advancements in surgical techniques, radiation, and chemotherapy, the five-year survival rate for oral cancer remains stagnant at about 50–55%. Early-stage oral cancer can masquerade as harmless lesions, making early detection critical for improving morbidity and mortality rates [8].

Several diagnostic methods exist for early oral cancer detection, including clinical examination and cytological studies of oral cells. Oral exfoliative cytology, a simple and non-invasive technique, is well-accepted by patients and effective for early diagnosis of oral cancer, including epithelial atypia and SCC. Early detection could prevent 80% of deaths, as 60% of oral cancers are advanced by the time of diagnosis. In the early stages, oral cancer appears as an innocent lesion, often leading to delayed reporting and more invasive treatments. Exfoliative cytology offers a straightforward, non-invasive diagnostic tool for early detection of premalignant and malignant oral lesions, potentially improving survival and morbidity rates. Timely diagnosis and cessation of Naswar/snuff use could revert the affected mucosa to a normal state [9, 10].

This study aims to determine the cytomorphological changes in oral mucosal cells among smokeless tobacco users and examine the association with gender and age groups.

## Materials and methods

### Study design and setting

A cross-sectional study was conducted at Sardar Begum Dental Hospital, Peshawar, Khyber Pakhtunkhwa, Pakistan, from November 2020 to January 2022, following approval from the Ethical Committee of the University of Gandhara (No:104 6th meeting).

### Participants

The study utilized a non-probability consecutive sampling technique. The sample consisted of 100 regular smokeless tobacco users (at least 5 years of Naswar/snuff use), with equal representation from both genders. Participants were aged 20–60 years. Exclusion criteria included cigarette smokers, alcoholics, users of other smokeless tobacco types (e.g., bidi, gutka, paan), individuals with systemic diseases (e.g., blood disorders, renal failure, diabetes mellitus), and those who had received radiotherapy and/or chemotherapy. Informed consent was obtained from all participants for both the procedure and the use of their data for research purposes.

### Data collection

Participants were instructed to rinse their mouths with water. Cytological smears were collected using a brush and wooden spatula from the area where naswar/snuff was placed. The smear was transferred to a dry glass slide immediately. The slides were fixed in 95% ethanol and stained with Papanicolaou (PAP) and May-Grunwald Giemsa (MGG) stains, each requiring different fixation methods. PAP stain required immediate fixation in 95% alcohol, while MGG stain required air-drying before fixation in methanol. The stained smears were then observed under 40X and 100X magnifications to identify dysplastic changes such as micronuclei, multinucleation or binucleation, nuclear budding, and perinuclear halo.

The collection of samples and staining of each slide was initially examined microscopically by the principal investigator, SH, and subsequently re-evaluated by WN. To address inter-examiner variability, we compared the evaluations made by both experts. In cases of discrepancies, a consensus was reached after joint review of the slides.

Despite our efforts to achieve comprehensive data collection, there were occasional instances of lost data due to incomplete procedures, such as missed appointments or participant dropouts. To mitigate this, we implemented a follow-up protocol to encourage participant engagement and collect any missing data whenever feasible. Additionally, we carefully documented all instances of lost data in our records. This approach helped ensure that our results remained robust and representative of the available information.

### Statistical analysis

Data were analyzed using SPSS Version 25. Continuous variables (age, duration) were presented as mean ± standard deviation (SD) while these were later on transformed to categorical variables. Categorical variables (gender, site, amount of Naswar/snuff used, duration of Naswar/snuff use, clinical presentation, and cytomorphological changes) were analyzed using the Chi-square test to determine the distribution of cytomorphological parameters among different independent variables. Multi-logistic regression was applied to check for associations and confounders to get an adjusted odds ratio (AOR) considering variables with significant *p*-values. A *p*-value ≤ 0.05 was considered statistically significant.

## Results

The study included 100 participants with a mean age of 45.89 ± 10.14 years, categorized into different age groups. Most participants were male (76%). The labial sulcus mucosa was the most common site for mucosal changes (47%), followed by the buccal sulcus mucosa (41%), with only 12% involving the floor of the mouth. Notably, 94% of participants used less than one pack of smokeless tobacco daily. The most frequent duration of smokeless tobacco use was 10–20 years (48%). Clinically, 65% of users had a reddened or yellowish-white wrinkled appearance of the mucosa. The most common cytomorphological changes observed were micronuclei (46%), followed by nuclear budding (25%), perinuclear halo (18%), and binucleated nuclei (14%) (Table 1).

**Table 1:** Demographic Characteristics of Smokeless Tobacco Users

Variables	Categories		Frequency		Percentage	
Age	20–30 years		11		11	
	31–40 years		23		23	
	41–50 years		29		29	
	51–60 years		37		37	
Gender	Male		76		76	
	Female		24		24	
Site of smokeless tobacco placement	Labial sulcus		47		47	
	Buccal sulcus		43		43	
	Floor of the mouth		10		10	
Amount of smokeless tobacco	< 1 pack		94		94	
	> 1 pack		6		6	
Duration of smokeless tobacco	10–20 years		48		48	
	21–30 years		30		30	
	31–40 years		22		22	
Clinical presentation	Normal colouration with slight wrinkled mucosa		25		25	
	Reddened or yellowish white wrinkled mucosa		65		65	
	Heavily wrinkled, thickened, deep reddened furrows		10		10	
Cytomorphological changes	Micronuclei		46		46	
	Nuclear budding		25		25	
	Perinuclear halo		18		18	
	Bi nucleation		14		14	
Descriptive statistic of continuous variable like age and duration of naswar/snuff	Mean	Std. Deviation	Range	Minimum		Maximum
Age	45.89	10.147	40	20		60
Duration	21.16	7.091	25	10		35

Micronuclei were predominantly present in the 51–60 years age group (78.9%, *P* < 0.001), with no presence in the 20–30 years group. Similarly, nuclear budding was most common in the 51–60 years age group (55.3%, *P* < 0.001), with no presence in the 20–30 and 31–40 years groups. Gender-wise, micronuclei were more frequently reported in females (54.2%) compared to males (43.4%), with a *P* value of 0.35. In contrast, nuclear budding was more common in males (27.6%) than females, with a *P* value of 0.28 (Table 2).

**Table 2:** Distribution of Micronuclei and Nuclear Budding among Smokeless Tobacco Users by Age, Gender, Site, Amount, Clinical Presentation, and Duration

Variable	Cytomorphological Changes							
	**Micro Nuclei**			*P* value*	**Nuclear budding**			*P* value*
	Level	Present	Not Present		Level	Present	Not Present	
Age	20–30	0(0.0%)	10(100.0%)	< .001	20–30	0(0.0%)	10(100.0%)	< .001
	31–40	4(18.2%)	18(81.8%)		31–40	0(0.0%)	22(100.0%)	
	41–50	12(40.0%)	18(60.0%)		41–50	49(13.3%)	26(86.7%)	
	51–60	30(78.9%)	8(21.1%)		51–60	21(55.3%)	17(44.7%)	
Gender	Male	33(43.4%)	43(56.6%)	0.35	Male	21(27.6%)	55(72.4%)	0.28
	Female	13(54.2%)	11(45.8%)		Female	4(16.7%)	20(83.3%)	
Site	Labial	22(46.8%)	25(53.2%)	0.005	Labial	11(23.4%)	36(76.6%)	< .001
	Buccal	15(34.9%)	28(65.1%)		Buccal	6(14.0%)	37(86.0%)	
	Mouth floor	9(90.0%)	1(10.0%)		Mouth floor	8(80.0%)	2(20.0%)	
Amount	> 1 pack daily	44(46.8%)	50(53.2%)	0.68	> 1 pack daily	24(25.5%)	70(74.5%)	1.0
	< 1 pack daily	2(33.3%)	4(66.7%)		< 1 pack daily	1(16.7%)	5(83.3%)	
Clinical Picture	Normal	0(0.0%)	25(100.0%)	< .001	Normal	0(0.0%)	25(100.0%)	< .001
	Wrinkled	36(55.4%)	29(44.6%)		Wrinkled	16(24.6%)	49(75.4%)	
	Heavily wrinkled	10(100.0%)	0(0.0%)		Heavily wrinkled	9(90.0%)	1(10.0%)	
Duration	20–30	1(2.1%)	47(97.9%)	< .001	10_20	0(0.0%)	25(100.0%)	< .001
	21–30	23(76.7%)	7(23.3%)		21–30	16(24.6%)	49(75.4%)	
	31–40	22(100.0%)	0(0.0%)		31–40	9(90.0%)	1(10.0%)	

*P* vlue ≤ .05 was taken as significant

^*^Chi Square test/Fisher Exact Test

**Fig. 1:**
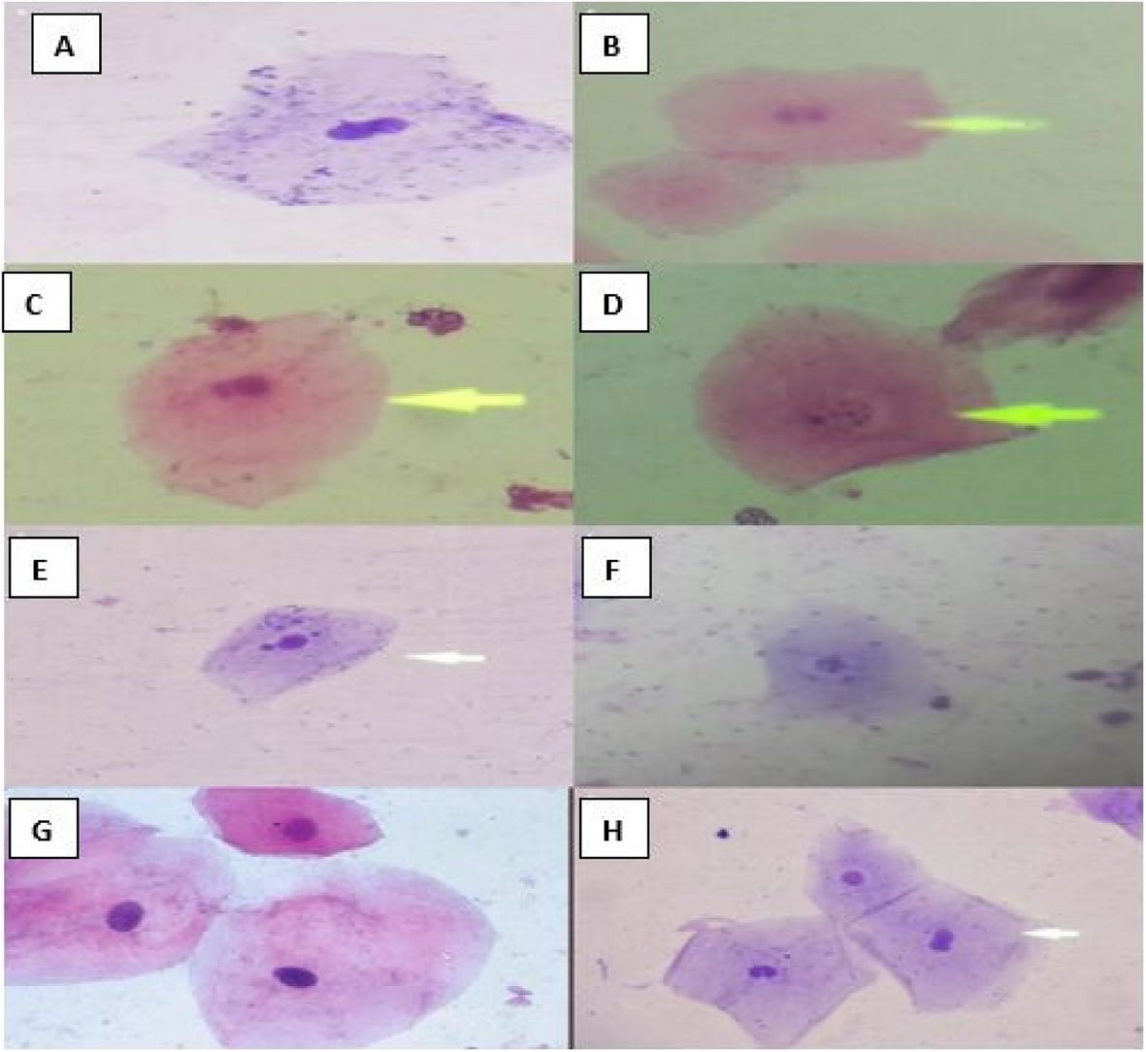
Cytomorphological changes of oral mucosa in snuff users. **A** Nuclear budding in a labial smear of snuff users (PAP stain), **B** & **C**. binucleation in a buccal smear of snuff users (PAP stain). **D** Perinuclear halo (indicative of HPV in labial smear (PAP stain), **E** Labial smear showing micronuclei (MGG stain), **F** Perinuclear halo (MGG stain) **G** Normal buccal mucosa smear (MGG stain), **H** Nuclear budding and binucleation in buccal smear (MGG stain)

The floor of the mouth was significantly more affected by both micronuclei (90%, *P* = 0.005) and nuclear budding (80%, *P* < 0.001). The labial sulcus mucosa showed a 46.8% presence of micronuclei (*P* = 0.005) and a 23.4% presence of nuclear budding (*P* < 0.001). There was no significant difference in micronuclei presence between users of more than one packet daily (46.8%) and those using less than one packet daily (33.3%) (*P* = 0.68). Similarly, there was no significant difference in nuclear budding related to the amount of daily usage (*P* = 1.0). Figure 1 demonstrates the distinctive cytomorphological changes observed in oral mucosa of snuff users, including nuclear budding, binucleation, perinuclear halo formation and micronuclei presence, as compared to normal buccal mucosa. These changes were observed using both PAP and MGG staining techniques.

Participants with wrinkled mucosa had a higher presence of micronuclei (55.4%, *P* < 0.001) and nuclear budding (24.6%, *P* < 0.001). Heavily wrinkled mucosa showed 100% presence of micronuclei and 90% presence of nuclear budding. Regarding the duration of use, micronuclei were significantly more present in participants with 31–40 years of smokeless tobacco use (100%, *P* < 0.001). Nuclear budding was most common in the 21–30 years group (76.7%, *P* < 0.001) (Table 3).

**Table 3:** Distribution of Binucleated Cells and Perinuclear Halo among Smokeless Tobacco Users by Age, Gender, Site, Amount, Clinical Presentation, and Duration

Variable	Cytomorphological Changes							
	**Bi Nucleated**			*P* value*	**Peri Nuclear Halo**			*P* value*
	Level	Present	Not Present		Level	Yes	No	
Age	20–30	0(0.0%)	10(100%)	0.001	20–30	0(0.0%)	10(100.0%)	0.002
	31–40	0(0.0%)	22(100%)		31–40	1(4.5%)	21(95.5%)	
	41–50	2(6.7%)	28(93.3%)		41–50	3(10.0%)	27(90.0%)	
	51–60	12(31.6%)	26(68.4%)		51–60	14(36.8%)	24(63.2%)	
Gender	Male	11(14.5%)	65(85.5%)	1.0	Male	14(18.4%)	62(81.6%)	1.3
	Female	3(12.5%)	21(87.5%)		Female	4(16.7%)	20(83.3%)	
Site	Labial	6(12.8%)	41(87.2%)	0.005	Labial	7(14.9%)	40(85.1%)	0.004
	Buccal	3(7.0%)	40(93.0%)		Buccal	5(11.6%)	38(88.4%)	
	Mouth floor	5(50.0%)	5(50.0%)		Mouth Floor	6(60.0%)	4(40.0%)	
Amount	> 1 pack daily	13(13.8%)	81(86.2%)	1.2	> 1 pack daily	17(18.1%)	77(81.9%)	1.0
	< 1 pack daily	1(16.7%)	5(83.3%)		< 1 pack daily	1(16.7%)	5(83.3%)	
Clinical Picture	Normal	0(0.0%)	25(100.0%)	< .001	Normal	0(0.0%)	25(100.0%)	< .001
	Wrinkled	5(7.7%)	60(92.3%)		Wrinkled	10(15.4%)	55(84.6%)	
	Heavily wrinkled	9(90.0%)	1(10.0%)		Heavily wrinkled	8(80.0%)	2(20.0%)1112(20.0%)	
Duration	10_20	0(0.0%)	48(100.0%)	< .001	10_20	0(0.0%)	48(100.0%)	< .001
	21–30	0(0.0%)	30(100.0%)		21–30	7(7(23.3%)	23(76.7%)	
	31–40	14(63.6%)	8(36.4%)		31–40	11(50.0%)	11(50.0%)	

*P* value ≤ .05 was taken as significant

^*^Chi Square test/Fisher Exact Test was applied

Logistic regression analysis in Table 4 revealed that participants in the 51–60 years age group were more likely to exhibit micronuclei, although the association was not statistically significant (AOR = 1.15, 95% CI: 0.23 to 5.83, *P* = 0.863). In contrast, participants in the 51–60 years age group were significantly more likely to show nuclear budding, with an odds ratio of 15.34 (95% CI: 9.23 to 30.75, *P* = 0.05).

**Table 4:** Multi Logistic Regression Analysis of Cytomorphological Changes in Smokeless Tobacco Users

Dependent variable	Independent variable	*P* value	AOR*	95% Confidence interval	
				Lower	Upper
**Micro Nuclei** Presence (Yes/No)	Ref Age(20–30)				
	31–40	0.13	0.29	0.058	1.44
	41–50	0.075	0.239	0.05	1.15
	51–60	0.863	1.153	0.228	5.83
	Site Ref(Labial)				
	Buccal	0.555	0.76	0.301	1.90
	Mouth floor	0.016	14.5	04.82	42.02
**Nuclear budding** Presence (Yes/No)	Ref Age(20–30)				
	31–40	0.482	1.902	0.316	11.44
	41–50	0.464	1.925	0.333	11.11
	51–60	0.05	15.34	9.23	30.75
	Site Ref(Labial)				
	Buccal	0.566	1.314	0.517	3.337
	Mouth floor	0.075	4.70	0.853	25.844
**Bi Nucleated** Presence (Yes/No)	Ref Age(20–30)				
	31–40	0.923	0.883	0.07	11.08
	41–50	0.876	0.825	0.073	9.31
	51–60	0.314	3.189	0.333	30.52
	Site Ref(Labial)				
	Buccal	0.663	0.755	0.213	2.671
	Mouth floor	0.152	3.002	0.666	13.522
	*Adjusted Odds Ratio				

## Discussion

The objective of this study was to determine the cytological changes in oral mucosal cells among smokeless tobacco (naswar/snuff) users, examining markers such as micronuclei, nuclear budding (broken-egg nuclei), binucleated cells, and perinuclear halo (indicative of HPV virus), and their distribution across gender and age groups. Our findings showed that 46% of cases exhibited micronuclei, 25% had nuclear buds, 14% had binucleated cells, and 18% had a perinuclear halo. Few studies have explored these aspects.

Smokeless tobacco consumption not only increases the chances of dental caries [11] and deterioration of periodontal health [12] but also causes cell abnormalities, for instance, binucleation and cellular atypia [13, 14]. Another study reported that the micronuclei count in smokeless tobacco users was higher than in non-smokers/non-users [15]. These findings align with other studies that showed an increased frequency of micronuclei in smokeless tobacco users compared to control groups [16–18]. Typical cellular changes among tobacco users, including nuclear enlargement indicative of HPV presence, were observed in some buccal smears [19]. Similarly, a local study reported a significant cellular and nuclear diameter increase among naswar/snuff users [20]. A study described "broken egg nuclei" and binucleated cells, noting that these cells result from chromosome aberrations and failure to complete mitosis [21]. Our observations of cytomorphological changes match previous studies, suggesting a common biological pathway in humans [22, 23].

Of the 100 participants, 76% of smokeless tobacco users were male, and 24% were female. Most users were in their 5th and 6th decades (51–60 years), with a mean age of 38 years (SD ± 2.34). A study revealed that about 15% of males and 10% of females aged 25–64 years regularly used chewing tobacco or snuff [24]. This demographic trend may be due to similar social and cultural factors, highlighting that our society is male-dominated, with easy access to naswar/snuff.

Our results indicate that the most common site for naswar/snuff placement was the labial mucosa (47%), followed by the buccal mucosa (41%), with the floor of the mouth being the least common site. This contrasts with a study conducted in the same geographical which suggested that buccal mucosa as the most common site for smokeless tobacco placement [12]. However, in our study, local smokeless tobacco users believed the labial sulcus played a significant role in sedation, making it the most common site.

Ninety-four percent of participants used less than one pack daily, with 48% having used smokeless tobacco for 10–20 years. These results align with a study of 40 snuff users, where 31 participants used less than one packet per day, seven used one packet per day, and two used more than one packet per day [20]. Our study's setting was private sector, where high socioeconomic individuals seek treatment and have higher education levels, which may explain the lower frequency of naswar/snuff use among highly educated individuals.

Oral mucosal changes due to snuff usage are well-documented [25–27]. It is known that snuff causes damage to the oral cavity, resulting in thick, wrinkled, and discolored mucosa at the site of placement [14, 28]. In our study, 25% of smokeless tobacco users had normal coloration with slight wrinkling, 65% had reddened or yellowish-white wrinkled mucosa, and 10% had heavily wrinkled, thickened mucosa with deep, reddened furrows. It is assumed that longer naswar/snuff use leads to more apparent mucosal changes.

### Strengths and weaknesses

This study presents several strengths, including its innovative focus on the cytological changes induced by smokeless tobacco (naswar/snuff) on oral mucosal cells. Utilizing exfoliative cytology, a non-invasive technique, allows for early detection of potential malignancies, which is patient-friendly and practical for clinical settings. The study's specific focus on a population in Khyber Pakhtunkhwa, Pakistan, provides localized insights into regional practices and their health impacts, contributing valuable information for targeted public health interventions.

However, several weaknesses should be noted. Firstly, the study did not conduct a formal sample size calculation, which could affect the statistical power and generalizability of the findings. The use of non-probability sampling and recruitment from a private dental hospital limits the generalizability to the broader population. Additionally, the cross-sectional design precludes longitudinal assessment of the long-term effects of naswar/snuff use on the oral mucosa. Furthermore, while necessary, the exclusion criteria may not fully account for all potential confounding factors, such as variations in diet and oral hygiene practices, which could influence the study outcomes. Lastly, the study's scope was limited to cytological changes without investigating potential molecular or genetic alterations associated with naswar/snuff use.

## Conclusion

Smokeless tobacco use, specifically habitual naswar/snuff chewing, can cause significant changes in the oral mucosa, detectable through exfoliative cytology. The cytological changes observed in this study included micronuclei, nuclear budding (broken eggs), binucleation, and perinuclear halo. These changes were prevalent among men, particularly in their 5th and 6th decades of life. The labial mucosa was the most common site for naswar/snuff placement, followed by the buccal mucosa, with varied clinical presentations. To better understand these effects and confirm these findings, further studies with larger sample sizes, standardized parameters, and advanced techniques are needed.

### Recommendation

Oral cancers are often preceded by precursor lesions, which can greatly aid in early diagnosis. While naswar/snuff may not be as harmful as smoking cigarettes, it is important to recognize that all forms of tobacco carry significant health risks, including the potential for oral cancer. Exfoliative cytology, a simple and non-invasive diagnostic technique, can be an effective tool for the early detection of dysplastic changes in oral lesions. Therefore, we recommend implementing routine cytological screening for all naswar/snuff users to facilitate early identification and intervention.

## Data Availability

Data is provided within the manuscript.
