# Correction: Cytomorphological changes of oral mucosal cells among smokeless tobacco users in low and middle-income country settings: new findings from Pakistan

**DOI:** 10.1186/s12903-025-05710-2

**Published:** 2025-02-27

**Authors:** Sabreen Hassan, Ayesha Sifat, Mohammad Munib, Saleha Saeed, Waqar Un Nisa, Sofia Haider Durrani, Abid Rahim, Naeem Ullah, Saima Afaq, Farhad Ali Khattak, Zia Ul Haq

**Affiliations:** 1 Saidu College of Dentistry, Swat, Pakistan; 2 Oral Pathology Department, Saidu College of Dentistry, Saidu Sharif, Swat, Pakistan; 3 Khyber Medical University, Peshawar, Pakistan; 4 Swat Medical College, Swat, Pakistan; 5 Dental College, HITEC– IMS, Taxila, Pakistan; 6 Bacha Khan Dental College, Mardan, Pakistan; 7 Oral Pathology Department, Bacha Khan College of Dentistry, Mardan, Pakistan; 8 Oral Pathology Department, Sardar Begum Dental College, Gandhara University, Peshawar, Pakistan; 9 Gandhara University, Peshawar, Pakistan; 10 Saidu Medical College, Swat, Pakistan; 11 Departmentof Health Sciences, University of York, York, UK; 12 Institue of Public Health & Social Sciences(IPH&SS), Khyber Medical University(KMU), Peshawar, Pakistan; 13 R&D Cell, Khyber College of Dentistry, Peshawar, Pakistan; 14 School of Health & Wellbeing, University of Glasgow, Glasgow, Scotland, UK

**Correction to: BMC Oral Health (2024) 24:1541**

10.1186/s12903-024-05220-7.

In this article [1], the author’s name Waqar Un Nisa was incorrectly written as Waqar U. Nisa.
